# Effects of high-intensity resistance training on extended body composition and functional fitness after spinal cord injury with motor complete paraplegia: a randomized controlled trial study

**DOI:** 10.3389/fpubh.2025.1678313

**Published:** 2025-10-30

**Authors:** Seckjin Kim, Junmin Lee, Wonjung Kim, Seungmo Jin, Younghyeon Bae, Hyunjong Lee, Junghwan Kim, Kyungjun An, Nohhwan Park, Seyoung Shin

**Affiliations:** ^1^Department of General Affairs, National Rehabilitation Center, Seoul, Republic of Korea; ^2^Department of Gaitlab, National Rehabilitation Center, Seoul, Republic of Korea; ^3^Department of Physical Education, Konkuk University, Seoul, Republic of Korea; ^4^National Team Physical Fitness Assessment and Analysis Lab, Department of Sport Science, Sport Science Center for National Athletes with Disabilities, Icheon, Republic of Korea; ^5^Department of Healthcare and Public Health, Rehabilitation Research Institute, National Rehabilitation Center, Seoul, Republic of Korea; ^6^Department of Clinical Research for Rehabilitation, Rehabilitation Research Institute, National Rehabilitation Center, Seoul, Republic of Korea; ^7^Department of Rehabilitation Medicine, Ewha Womans University Mokdong Hospital, Seoul, Republic of Korea; ^8^Department of Sports and Leisure, Suseong University, Daegu, Republic of Korea; ^9^Department of Physical Education, Korea National Sport University, Seoul, Republic of Korea

**Keywords:** neurological impairment, disability, resistance exercise, health promotion, rehabilitation training

## Abstract

**Purpose:**

This study aimed to compare the effects of high-intensity resistance training (HIRT) versus moderate-intensity resistance training (MIRT) on bone mineral density (BMD), body composition, and functional fitness in individuals with motor-complete paraplegia after spinal cord injury (SCI), and to propose a tailored exercise intervention for this population.

**Methods:**

Participants with motor-complete paraplegia were randomized into HIRT (*n* = 8) or MIRT (*n* = 8) groups. Both groups completed an 8-week elastic resistance training program. The measured outcomes included extended body composition (BMD, T-scores, lean mass, and fat mass) and functional fitness components (cardiorespiratory endurance, muscular strength, endurance, and flexibility).

**Results:**

No significant changes in BMD were observed in either group (*p* > 0.05). The HIRT group demonstrated significant improvements in lean mass (*p* < 0.001), chest press strength (*p* = 0.024), muscular endurance (*p* = 0.008), and VO₂peak (*p* = 0.001), while the MIRT group showed no significant changes. Flexibility and fat mass did not significantly differ in either group (*p* > 0.05).

**Conclusion:**

High-intensity resistance training was more effective than MIRT in improving lean mass and functional fitness in individuals with motor-complete SCI. Although BMD did not change over the 8-week period, its assessment remains clinically relevant, and future studies should investigate longer-duration or higher-intensity protocols to promote skeletal adaptations.

## 1 Introduction

Individuals with spinal cord injury (SCI) represent a growing demographic, with over 2,000 new cases reported annually across all age groups and sexes (1). Due to high healthcare costs and susceptibility to secondary conditions, tailored health programs are essential (2). Loss of motor function in SCI leads to reduced physical activity, which is a primary contributor to poor health outcomes. Owing to their dependence on assistive devices such as wheelchairs, individuals with SCI exhibit significantly lower physical activity levels than those without disabilities. They also show markedly poorer physical fitness, including reduced muscular strength, flexibility, and cardiorespiratory endurance.

In individuals with SCI, cardiorespiratory endurance is approximately 70% of the typical level, while muscular strength ranges from 30 to 50% depending on injury severity (3). Flexibility is often limited by repetitive, constrained movements during wheelchair use, which increases the risk of musculoskeletal injury (4). Thus, maintaining and improving physical fitness is essential for preserving independence and overall health and requires continuous monitoring and effort. Achieving this necessitates the consideration of extended body composition, including bone mineral density (BMD) and body composition, alongside functional fitness, cardiorespiratory endurance, muscular strength, muscular endurance, and flexibility. Given the heightened risk of osteoporosis and fracture in SCI, BMD was included as a clinically meaningful component of extended body composition analysis, providing insights into long-term skeletal health beyond immediate effects of training.

Reduced activity in SCI increases the risk of osteoporosis and fractures (5). However, regular upper-body exercise may enhance bone health, with some studies reporting increased upper-limb BMD in active individuals with SCI (6). There is a strong relationship between physical fitness and BMD. Regular exercise promotes muscle fiber development and stimulates the musculoskeletal system, thereby improving BMD. High-intensity exercise, which imposes a greater mechanical load, can induce hypertrophy and enhance strength, leading to BMD gains in targeted areas. SCI affects the entire body, and interventions such as electrical stimulation, which induce repetitive contractions and mechanical stress, have been shown to improve BMD (7). Therefore, maintaining physical fitness is key to increasing BMD and improving the overall health of individuals with SCI.

Researchers continue to explore innovative training methods to maximize the effectiveness of exercise. One prime example is high-intensity interval training (HIIT), which has gained popularity for its time efficiency and high effectiveness. In the present study, the traditional aerobic-based HIIT and moderate intensity continuous training (MICT) formats were modified into resistance-based protocols using elastic bands, allowing participants to perform interval or continuous exercises with upper-limb muscle engagement rather than large-muscle aerobic movement. This approach was specifically designed to align with the functional needs and neuromuscular conditions of individuals with SCI.

A 12-week HIIT program improved muscular strength and cardiovascular and respiratory functions in patients with SCI (8). Compared with continuous moderate-intensity training, HIIT provides higher metabolic intensity and greater enjoyment, making it more suitable for this population (9). Although the exercise modality was resistance-based, the interval and continuous structures of HIIT and MICT were maintained, ensuring comparable intensity modulation and work-to-rest ratios consistent with conventional definitions. It also yields superior oxygen uptake and cardiovascular fitness, improving vascular and overall health (10). Nevertheless, most individuals with SCI still rely on arm ergometer-based MICT (11), Therefore, adapting both HIIT and MICT into resistance-based formats provides an opportunity to enhance muscular engagement and functional outcomes while preserving the established principles of exercise intensity control. This study aimed to evaluate the effects of High-Intensity Resistance Training (HIRT) and Moderate-Intensity Resistance Training (MIRT) on functional fitness and extended body composition in individuals with SCI, and to propose an effective training method tailored to their needs.

## 2 Materials and methods

### 2.1 Participants

This study was conducted in accordance with the Declaration of Helsinki and was approved by the Institutional Review Board (IRB) of Konkuk University (IRB No. 7001355-202008-HR-397). Data collection was conducted from October 1, 2020, to March 31, 2021. Participants with chronic SCI were recruited from local disability centers. Inclusion criteria were: (1) wheelchair users with motor-complete paraplegia due to SCI, based on findings that individuals with American Spinal Injury Association Impairment Scale (AIS) grade B benefit from both short- and long-term training at various intensities (12); (2) no engagement in regular exercise within the past 6 months; (3) no use of medications that could affect study outcomes and no plans to initiate such medications during the study period; and (4) ability to complete the exercise program consistently. All participants were informed of the study’s purpose, protocol, and potential benefits and provided written informed consent.

Based on two comparable randomized controlled trials that showed clinically meaningful improvements with 7–10 participants per arm (11, 13), together with prior exercise-intervention studies in individuals with SCI (14, 15), we set a target of eight participants per group and, after allowing for an anticipated 20% attrition, enrolled 20 men. An investigator not involved in outcome assessments then used sealed opaque envelopes and a block size of 4 to allocate participants to either HIRT or MIRT.

Four participants withdrew because of recurrent pressure ulcers (*n* = 2) or scheduling conflicts (*n* = 2), leaving 16 participants (HIRT = 8; MIRT = 8) who completed the program. Baseline characteristics and AIS classifications are presented in Tables 1, 2, respectively.

**Table 1:** Participants’ physical characteristics (*N* = 16).

Group (*n*)	Age	Height	Body weight	BMI	Duration of disability
HIRT (8)	45.63 ± 8.08	175.25 ± 6.13	73.75 ± 9.99	24.19 ± 4.43	12.50 ± 4.03
MIRT (8)	36.25 ± 4.74	172.01 ± 6.80	78.38 ± 12.58	26.66 ± 5.18	11.50 ± 3.78

HIRT, High-Intensity Resistance Training Group; MIRT, Moderate-Intensity Resistance Training Group.

**Table 2:** AIS grades and SCI levels of participants.

HIRT no	AIS	SCI level	MIRT no	AIS	SCI level
HIRT 1	B	T8 ~ T9	MIRT 1	B	T8
HIRT 2	B	T9	MIRT 2	B	T7 ~ T8
HIRT 3	B	T6 ~ T7	MIRT 3	B	T5 ~ T6
HIRT 4	B	T9	MIRT 4	B	T7 ~ T8
HIRT 5	B	T6 ~ T7	MIRT 5	B	T5
HIRT 6	B	T8 ~ T9	MIRT 6	B	T4 ~ T5
HIRT 7	B	T7 ~ T8	MIRT 7	B	T6 ~ T7
HIRT 8	B	T4 ~ T5	MIRT 8	B	T9

AIS, American Spinal Injury Association Impairment Scale; SCI, spinal cord injury; HIRT, High-Intensity Resistance Training Group; MIRT, Moderate-Intensity Resistance Training Group.

### 2.2 Procedure

**Figure 1:**
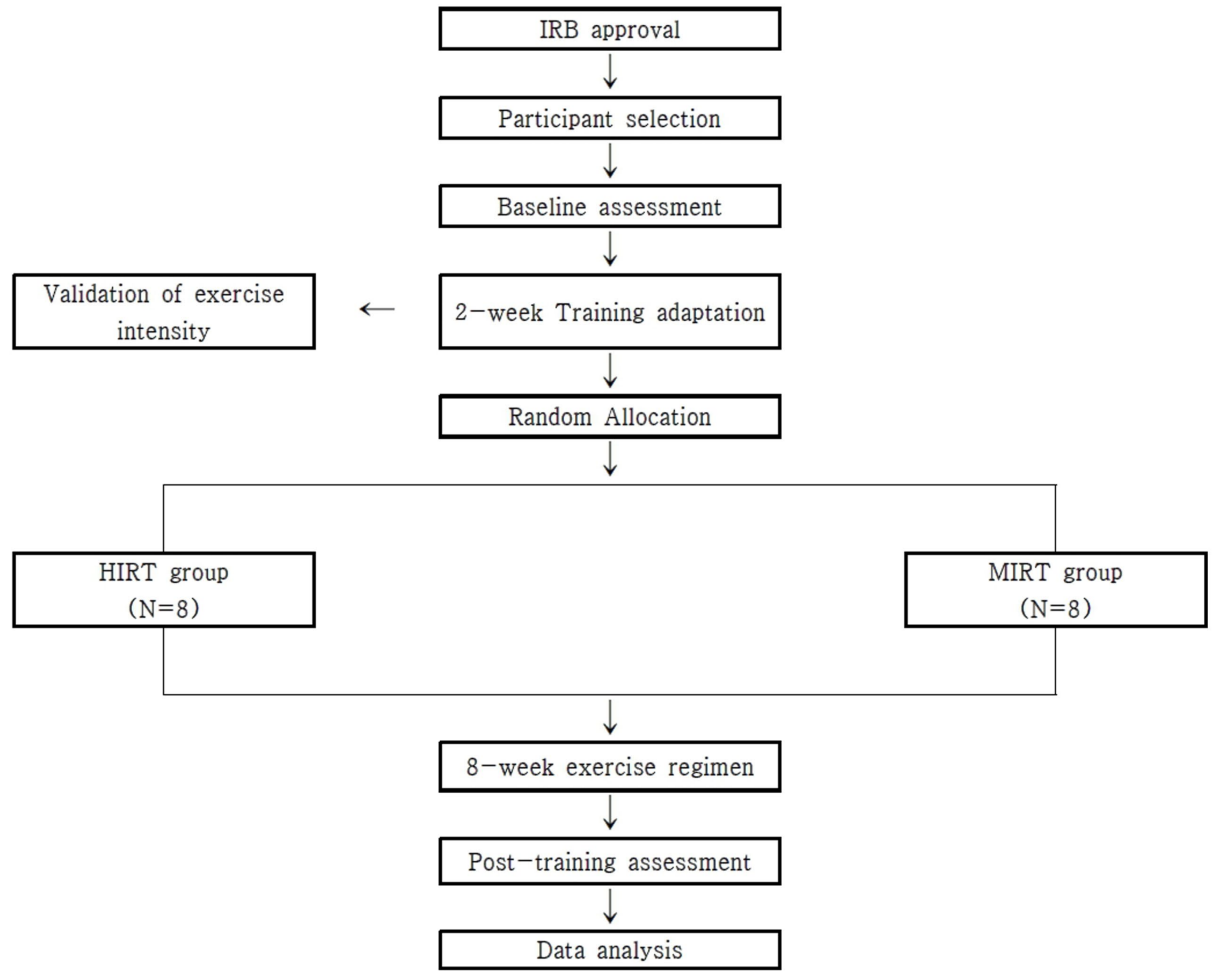
Study design.

This study investigated the differences in outcomes between training methods in individuals with SCI. Participants were assessed at baseline and randomly assigned to the HIRT or MIRT groups. After 8 weeks of training, the same protocols were used to assess changes in BMD and fitness. The study design is illustrated in Figure 1. Outcome assessors were blinded to group allocation throughout the study period to reduce assessment bias.

### 2.3 Training methods

This study implemented two training protocols, HIRT and MIRT, to examine their effects on individuals with SCI. Before the intervention, a pre-training validation phase was conducted to directly measure energy expenditure during MIRT sessions using a portable gas analyzer (K5, COSMED, Italy). These measurements served as the basis for adjusting the HIRT protocol to ensure equivalent training volume between groups. Based on a previous study indicating that at least two sessions of high-intensity exercise per week over 6 weeks are required to improve physical fitness in individuals with SCI (16), participants first completed a 2-week validation and acclimation phase, This phase consisted of once-weekly training to adjust to the prescribed intensities, provide instruction and practice for interpreting the rating of perceived exertion (RPE), and measure energy expenditure during MIRT sessions for protocol matching. The final decision to implement an 8-week intervention period was informed by previous studies demonstrating improvements in overall physical function among individuals with SCI following 8 weeks of exercise training (17, 18). Given the limited physical activity associated with daily life in this population, this duration was considered sufficient. To ensure that both external intensity (%1RM) and internal load (subjective exertion) were accurately represented, training sessions were standardized based on one-repetition maximum (1RM) to unify resistance intensity. During the intervention, researchers monitored RPE in real time and provided feedback to maintain adherence to the target intensity. Training was conducted three times per week, as research indicates that 2–3 weekly sessions of moderate- to high-intensity exercise improve strength and physical function in individuals with chronic SCI (19). A minimum of 24 h of rest was maintained between sessions to ensure adequate recovery. All exercises were designed according to the frequency, intensity, time, and type principle of exercise prescription. All training sessions were supervised by a rehabilitation exercise specialist with over 10 years of experience in SCI-specific physical training, ensuring safety and appropriate guidance throughout the intervention.

The HIRT protocol was adapted from a previous upper-body HIIT case study in an individual with SCI using an arm crank ergometer (20). It included high-intensity intervals at 70% of one repetition maximum (1RM) with a rating of perceived exertion of 17 on Borg’s scale, lasting 4–5 min, alternating with low-intensity intervals at 20–25% of 1RM for 3–5 min. In previous resistance training studies involving individuals with spinal cord injury, 70% of 1RM has been classified as high intensity (21), and the original HIRT design that guided this study also applied 70% as the high-intensity threshold (20). Based on these precedents, this study defined 70% of 1RM as high-intensity resistance training. Each interval consisted of 2 to 3 sets of 30–45 s, adjusted to match the energy expenditure measured during the MIRT validation phase. Resistance bands enabled smooth transitions between intensities, and repetitions were tailored to match the energy expenditure measured in MIRT.

The MIRT group performed resistance exercises continuously, without rest between repetitions or sets, maintaining a steady pace throughout the 25 min sessions. This structure was intentionally designed to reflect the principles of continuous training, despite the use of resistance-based rather than aerobic modalities. The MIRT protocol was developed based on previous research applying moderate-intensity continuous training to individuals with SCI. It consisted of 25 min sessions performed at 45% of 1RM, with participants maintaining a perceived exertion level of 12 on Borg’s scale. Each session lasted approximately 30 min in total, including a 3 min warm-up and 2 min cool-down phase with standardized stretching exercises. This structure was maintained consistently across both groups to ensure uniform physiological preparation and recovery. The total energy expenditure of these MIRT sessions was measured and used as a reference to ensure matched training volume in the HIRT protocol. Resistance bands were calibrated according to the 2019 guidelines of the National Rehabilitation Center (22). Training intensity was adjusted at week 4, following 1RM reassessments, to align with progressive overload principles. Researchers monitored perceived exertion levels throughout each session to ensure participants exercised at the prescribed intensity.

Each training week included three sessions with one resistance exercise per day—chest press (day 1), lateral raise (day 2), and shoulder press (day 3). This structure was applied identically across both groups. In the chest press, participants positioned the resistance band behind the scapula, pushing forward to engage the chest and arm muscles. The lateral raise involved placing the band under the feet while seated in a wheelchair, lifting the hands to shoulder height to activate the shoulder muscles. The shoulder press required securing the band at the pelvic area in the wheelchair, either by sitting on it or looping it under the seat, and extending the arms upward.

To enhance adherence and accessibility, all sessions were conducted remotely at fixed times through a social network platform. Participants performed exercises simultaneously from their homes or workplaces according to pre-announced schedules. Real-time video monitoring ensured proper execution, and participants who missed a session were offered make-up opportunities.

To control for confounding variables, training volume was analyzed based on energy expenditure and total oxygen consumption. An independent t-test confirmed no significant differences between the groups (*t* = 0.166, *p* = 0.869), indicating equivalent training volumes. Detailed statistical results are presented in Table 3.

**Table 3:** Comparison of training volume between the two training methods.

Training method (sessions)	Energy expenditure (kcal)	*t*	*p*
HIRT (16)	129.11 ± 53.24	0.166	0.869
MIRT (16)	126.15 ± 47.46		

HIRT, High-Intensity Resistance Training Group; MIRT, Moderate-Intensity Resistance Training Group.

### 2.4 Outcome measures

#### 2.4.1 Extended body composition

Extended body composition, including BMD and body composition, was measured using dual-energy X-ray absorptiometry (Holyzonwi, United States). Scans were conducted at a certified facility with minimal radiation exposure (0.001 mSv) under professional supervision. Participants wore lightweight clothing and removed all metallic items. BMD and *T*-scores were assessed at the hip and lumbar spine, which are common sites of osteoporotic fractures (23). To ensure accuracy, the participants remained still during the scans. Body composition was recorded in grams and converted to kilograms.

#### 2.4.2 Functional fitness

Functional fitness was evaluated across cardiorespiratory endurance, muscular strength, muscular endurance, flexibility, and body composition.

Cardiorespiratory endurance was assessed using the 6 Minute Wheelchair Ambulation Test on an 80-m marked course. Participants were instructed to cover as much distance as possible within 6 min while using their wheelchair. The total distance traveled and VO₂ peak were recorded using a portable gas analyzer (K5, COSMED, Italy). All participants received prior instruction and completed a warm-up to ensure maximal effort during the test.

Muscular strength was assessed with an isometric chest press using a device (HUR, Finland). Before testing, participants received instruction and demonstration to ensure proper technique and maximal voluntary effort. Each participant performed a single maximal contraction, and the peak value was recorded for analysis.

Muscular endurance was evaluated with the 1 min arm curl test at 60% of the individual’s pre-assessed 1RM. While seated, participants performed as many repetitions as possible within 1 min, following a consistent range of motion under verbal supervision.

Flexibility was assessed with the behind-the-back reach test to evaluate shoulder mobility. The shortest distance between the fingertips of both hands was measured using a standard measuring tape. The test was conducted on both the affected and unaffected sides. When both sides were impaired, the more functional or less symptomatic side was classified as unaffected.

A trained safety officer was present during all assessments, and all researchers involved had received prior safety training to ensure standardized measurement procedures and participant safety.

### 2.5 Statistical analysis

Data were analyzed using SPSS version 28.0 (IBM Corp., Armonk, NY, United States) to compute means and standard deviations. The Shapiro–Wilk test confirmed a normal distribution across all groups and time points (*p* > 0.05), validating the use of parametric tests. Its appropriateness for small samples supports this choice.

Two-way repeated measures analysis of variance was used to examine the effects of training group (HIRT vs. MIRT) and time (pre- vs. post-intervention) on BMD and physical fitness. Within-group changes were analyzed using a paired *t*-test. Statistical significance was set at *p* < 0.05. Per-protocol analysis was conducted, excluding participants who withdrew before post-testing. No imputation methods were applied for missing data.

## 3 Results

### 3.1 Participants’ physical characteristics

The Shapiro–Wilk test confirmed a normal distribution in the selected sample (*p* > 0.05). Due to irregular attendance or health issues, the final sample comprised 16 participants who completed either the HIRT or MIRT program. Physical characteristics are presented in Table 1, and AIS classifications are shown in Table 2. No statistically significant differences were observed between groups in age, height, body weight, body mass index, or duration of disability (*p* > 0.05) (Table 1).

Participants in both groups were classified as AIS grade B and presented with comparable levels of SCI, with neurological injury levels ranging from T4 to T9 (Table 2). No apparent differences in AIS classification or neurological level were observed between groups.

Training volume was monitored using energy expenditure metrics to ensure equivalent load across intervention types. Analysis revealed no statistically significant difference in mean energy expenditure between the HIRT and MIRT groups (*p* = 0.869), confirming comparability in training volume (Table 3).

### 3.2 Extended body composition

A significant group-by-time interaction was observed for lean body mass (*p* < 0.05), along with a significant main effect of time (*p* < 0.01). The HIRT group demonstrated a significant post-training increase in lean mass (*p* < 0.001), whereas the MIRT group exhibited no change (*p* > 0.05) (Table 4).

**Table 4:** Analysis of extended body composition.

Variable	Group	Pre	Post	*t*	*p*		*F*	*p*
BMD (Hip) (g/cm^2^)	HIRT	0.75 ± 0.19	0.76 ± 0.17	−0.774	0.465	T	0.708	0.414
						G	0.510	0.487
	MIRT	0.81 ± 0.12	0.81 ± 0.12	−0.420	0.687			
						I	0.058	0.813
*T*-score (Hip)	HIRT	−1.36 ± 1.35	−1.28 ± 1.26	−1.024	0.340	T	0.986	0.338
						G	0.403	0.536
	MIRT	−0.99 ± 0.88	−0.95 ± 0.87	−0.406	0.697			
						I	0.158	0.697
BMD (Lumbar) (g/cm^2^)	HIRT	1.12 ± 0.26	1.10 ± 0.19	0.562	0.592	T	0.045	0.834
						G	0.208	0.655
	MIRT	1.16 ± 0.22	1.17 ± 0.23	−1.445	0.192			
						I	0.774	0.364
*T*-score (Lumbar)	HIRT	0.86 ± 2.16	0.67 ± 1.58	0.577	0.582	T	0.061	0.809
						G	0.173	0.683
	MIRT	1.09 ± 1.78	1.20 ± 1.89	−1.515	0.174			
						I	0.778	0.393
Lean fat mass (kg)	HIRT	45.59 ± 6.64	47.22 ± 6.44	−7.478	0.000^***^	T	9.991	0.007^**^
						G	0.245	0.628
	MIRT	44.60 ± 7.61	44.77 ± 7.13	−0.326	0.754			
						I	6.547	0.023^*^
Body fat mass (kg)	HIRT	27.43 ± 7.16	27.45 ± 8.16	−0.044	0.966	T	0.002	0.991
						G	0.777	0.393
	MIRT	30.94 ± 8.53	30.91 ± 7.82	0.054	0.959			
						I	0.005	0.946
Percent body fat (%)	HIRT	36.17 ± 7.09	35.53 ± 7.49	1.816	0.112	T	0.808	0.384
						G	1.11	0.310
	MIRT	39.21 ± 5.80	39.23 ± 5.01	−0.043	0.967			
						I	0.945	0.348

HIRT, High-Intensity Resistance Training Group; MIRT, Moderate-Intensity Resistance Training Group; BMD, bone mineral density, training; T, time; G, group; I, interaction. *p < 0.05; **p < 0.01; ***p < 0.001.

In contrast, no significant changes were observed in fat mass or percent body fat (*p* > 0.05). Similarly, no significant group-by-time interaction or main effects were found for hip BMD or *T*-scores (*p* > 0.05). Lumbar BMD and *T*-scores also showed non-significant results across all effects (*p* > 0.05) (Table 4).

### 3.3 Functional fitness

Functional fitness outcomes showed varied responses across domains. In muscular strength, chest press results revealed no significant group-by-time interaction or main effects (*p* > 0.05). However, paired t-tests indicated a significant within-group improvement in the HIRT group (*p* < 0.05), whereas no significant change was observed in the MIRT group (*p* > 0.05) (Table 5).

**Table 5:** Analysis of chest press (unit: kg) on the affected and non-affected sides.

Variable	Group	Pre	Post	*t*	*p*		*F*	*p*
Chest press (affected side) (kg)	HIRT	44.58 ± 12.74	58.77 ± 24.22	−2.869	0.024^*^	T	2.477	0.138
						G	0.339	0.570
	MIRT	45.63 ± 18.26	49.09 ± 17.32	−0.344	0.741			
						I	0.915	0.355
Chest press (unaffected side) (kg)	HIRT	52.29 ± 18.25	56.05 ± 16.35	−3.281	0.013^*^	T	0.366	0.555
						G	0.993	0.336
	MIRT	45.52 ± 20.07	47.92 ± 17.51	−0.237	0.819			
						I	0.018	0.896

HIRT, High-Intensity Resistance Training Group; MIRT, Moderate-Intensity Resistance Training Group; T, time; G, group; I, interaction. *p < 0.05.

For muscular endurance, on the affected side, a significant main effect of time was found (*p* < 0.01), though no interaction or group effects were observed (*p* > 0.05). Only the HIRT group demonstrated a significant within-group improvement (*p* < 0.01). On the unaffected side, a significant main effect of time was also observed (*p* < 0.05); however, neither group showed significant changes in within-group comparisons (*p* > 0.05) (Table 6).

**Table 6:** Arm curl (unit: times) in HIRT and MIRT groups.

Variable	Group	Pre	Post	*t*	*p*		*F*	*p*
Arm curl (affected side) (kg)	HIRT	40.25 ± 11.99	53.25 ± 13.87	−3.651	0.008^**^	T	9.717	0.008^**^
						G	0.001	0.976
	MIRT	44.25 ± 12.15	49.63 ± 15.70	−1.144	0.290			
						I	1.673	0.217
Arm curl (unaffected side) (kg)	HIRT	42.63 ± 13.75	51.13 ± 17.47	−1.936	0.094	T	6.206	0.026^*^
						G	0.115	0.739
	MIRT	44.88 ± 10.06	53.38 ± 17.13	−1.627	0.148			
						I	0.002	0.996

HIRT, High-Intensity Resistance Training Group; MIRT, Moderate-Intensity Resistance Training Group; T, time; G, group; I, interaction. *p < 0.05; **p < 0.01.

In terms of cardiorespiratory endurance, the 6 Minute Wheelchair Ambulation Test revealed a significant time effect (*p* < 0.01), with only the HIRT group showing significant post-training improvement (*p* < 0.05), while the MIRT group showed no change (*p* > 0.05). VO₂peak results demonstrated a significant group-by-time interaction (*p* < 0.05) along with a robust time effect (*p* < 0.001). Both groups improved significantly after training (HIRT: *p* < 0.01; MIRT: *p* < 0.05) (Table 7). Finally, flexibility measures showed no significant effects on either side (*p* > 0.05) (Table 8).

**Table 7:** Comparison of cardiorespiratory endurance (unit: m, ㎖/kg/min) in HIRT and MIRT groups.

Variable	Group	Pre	Post	*t*	*p*		*F*	*p*
6MWT (m)	HIRT	564.38 ± 165.13	652.13 ± 143.71	−2.710	0.030^*^	T	10.856	0.005^**^
						G	0.107	0.749
	MIRT	568.38 ± 146.59	599.50 ± 155.62	−1.956	0.091			
						I	2.463	0.139
VO_2_ peak (ml/kg/min)	HIRT	17.90 ± 5.18	25.02 ± 6.52	−4.147	0.004^**^	T	22.828	0.000^***^
						G	0.962	0.343
	MIRT	17.91 ± 4.83	20.09 ± 4.82	−2.376	0.049^*^			
						I	6.412	0.024^*^

HIRT, High-Intensity Resistance Training Group; MIRT, Moderate-Intensity Resistance Training Group; T, time; G, group; I, interaction; 6MWT, 6 Minute Walk Test. *p < 0.05; **p < 0.01; ***p < 0.001.

**Table 8:** Flexibility (unit: cm) of the affected and unaffected sides.

Variable	Group	Pre	Post	*t*	*p*		*F*	*p*
Flexibility (affected side) (cm)	HIRT	−23.37 ± 9.91	−21.25 ± 8.44	−0.959	0.369	T	1.158	0.300
						G	0.713	0.413
	MIRT	−30.62 ± 14.15	−26.00 ± 24.40	−0.788	0.456			
						I	0.159	0.696
Flexibility (unaffected side) (cm)	HIRT	−17.87 ± 10.48	−17.06 ± 13.54	−0.537	0.608	T	0.026	0.875
						G	0.162	0.693
	MIRT	−20.12 ± 16.34	−20.375 ± 15.90	0.079	0.939			
						I	0.091	0.767

HIRT, High-Intensity Resistance Training Group; MIRT, Moderate-Intensity Resistance Training Group; T, time; G, group; I, interaction.

## 4 Discussion

The BMD in SCI is affected by physical inactivity, increased adiposity, and impaired nutrient absorption (24, 25). Regular physical activity remains one of the most effective strategies for improving BMD (26). Mechanical loading through repeated muscle contractions and relaxations generates stress on bones, which increases the load borne by the skeletal system. This process minimizes bone resorption and helps maintain or improve BMD (27). Individuals with SCI, however, typically have reduced BMD compared to those without SCI, predisposing them to a higher risk of osteoporosis and fractures (28). For this population, consistent exercise is critical for preserving good BMD.

No significant change in BMD (*p* > 0.05) was observed over the 8-week intervention, likely reflecting the need for longer durations. In this study, BMD was assessed at the hip and lumbar spine, which are among the most fracture-prone sites in individuals with SCI and are clinically relevant for monitoring skeletal health. Although upper extremity BMD was not included, the selected regions represent key weight-bearing sites and are commonly used as proxies for overall skeletal status in clinical practice. Furthermore, given the high prevalence of osteoporotic fractures in the hip and spine among individuals with motor-complete SCI, as noted in our methodology, the study prioritized these regions to ensure both clinical relevance and consistency with prior SCI-related BMD research. Previous studies suggest that measurable osteogenic adaptations in individuals with SCI require at least 12 months of sustained high intensity loading (29, 30). Future research employing longer interventions, potentially combined with nutritional or pharmacological support, may further clarify the capacity of for exercise to promote measurable skeletal adaptations in SCI.

Exercise intensity also plays a crucial role. Studies suggest that intensity may have a greater impact than frequency or modality on promoting bone metabolism (31). Moderate-to high-intensity resistance training increases growth hormone secretion and osteoblast activity, thereby enhancing bone strength (32, 33). Although the intensity was individualized in this study, future protocols should incorporate progressive overload to better induce musculoskeletal adaptation.

The HIRT participants showed significant increases in lean mass (*p* < 0.001), which, while not directly improving BMD in the current timeframe, may contribute to future skeletal integrity by increasing axial loading and promoting muscle-bone interaction—critical precursors for long-term osteogenesis in SCI. According to a previous study (34), regular exercise in individuals with SCI resulted in significant increases in muscle mass. Such changes benefit not only BMD but also metabolic health and functional independence, especially in those reliant on upper-body strength. By contrast, the lack of lean mass improvement in the MIRT group may be partly attributed to the relatively low intensity of the resistance component, which was likely insufficient to induce hypertrophic adaptations.

The muscular strength and endurance gains in the HIRT group likely resulted from hypertrophic adaptation to high-intensity training. These findings align with prior research showing that hypertrophy improves force output, delays fatigue, and enhances physical performance (35). Muscle gains may also underlie improvements in oxygen uptake and cardiorespiratory fitness, consistent with evidence that resistance and interval training increase capillary density and cardiac output (36).

Despite these benefits, fat mass did not significantly decrease in either group, supporting previous findings that short-term resistance training alone has limited effects on body fat, unless combined with diet or extended duration (37). Similarly, 6 weeks of moderate-to-high intensity interval training improved strength metrics but did not significantly alter fat mass (13). Programs integrated with nutritional strategies may be required for favorable changes in fat composition.

Flexibility results were similarly limited, likely due to repetitive upper-body training and wheelchair-related biomechanical constraints (4). Muscle tightness from upper limb dominance underscores the need for adjunct flexibility or mobility interventions. Resistance training alone may be insufficient for improving joint range of motion, highlighting the value of posture- and flexibility-focused programs.

While HIRT yielded modest gains in body composition, its superior time efficiency, approximately 40% greater than that of MIRT, makes it a practical and scalable option for individuals with SCI (38). Given their time and physical limitations, HIRT provides an efficient model for enhancing fitness. Therefore, careful monitoring of intensity progression and recovery is essential to ensure safety and long-term adherence, especially in populations with limited mobility and autonomic regulation. Furthermore, incorporating behavioral support strategies and adaptive equipment may enhance program accessibility and support sustainable engagement in everyday environments, aligning with community-based physical activity goals. These findings provide actionable insights for designing inclusive, time-efficient training strategies that address both physiological and environmental barriers to exercise in the SCI population.

In conclusion, this study suggests that HIRT effectively improves lean mass, muscular strength, endurance, and cardiorespiratory fitness in individuals with SCI. While flexibility may require joint-specific or postural interventions, the overall musculoskeletal improvements observed in the HIRT group suggest potential benefits for long-term functional independence and health. Given the importance of lean mass in reducing the risks associated with metabolic syndrome and osteoporosis, HIRT emerges as a time-efficient and scalable rehabilitation strategy. Moreover, its feasibility within limited timeframes makes it a promising option for promoting community-based physical activity and improving health outcomes in populations with mobility impairments. Although the sample size was modest, it reflects the inherent challenges of recruiting individuals with motor-complete SCI into controlled training trials, and the findings nevertheless provide clinically meaningful insights for this underserved population.

## Data Availability

The raw data supporting the conclusions of this article will be made available by the authors, without undue reservation.
